# Teaching cornerball: a didactic proposal based on the sport education model

**DOI:** 10.3389/fspor.2026.1784916

**Published:** 2026-03-02

**Authors:** Pelayo Diez-Fernández, Alberto Rodríguez-Cayetano, Sandra Gutiérrez-Gutiérrez, Martín Barcala-Furelos, Iván González-Gutiérrez, Brais Ruibal-Lista, Sergio López-García

**Affiliations:** 1 Department of Functional Biology, Faculty of Medicine and Health Sciences, Universidad de Oviedo, Oviedo, Spain; 2 Grupo de Investigación en Actividad Física, Deporte y Salud (GIADES), Faculty of Education Universidad Pontificia de Salamanca, Salamanca, Spain; 3 Faculty of Education, Universidad Pontificia de Salamanca, Salamanca, Spain; 4 Faculty of Educational Sciences, Universidad de Santiago de Compostela, Santiago de Compostela, Spain; 5 Faculty of Social Sciences and Humanities, Universidad Europea del Atlántico, Santander, Spain; 6 EUM Fray Luis de León, Universidad Católica de Ávila, Valladolid, Spain

**Keywords:** alternative sports, educational innovation, motivation, physical education, secondary education

## Abstract

In response to the educational demands of the 21st century, Physical Education (PE) has evolved toward a more comprehensive model, requiring innovative approaches from teachers. Educational sport remains essential; however, excessive competitiveness in some traditional sports often fails to motivate students. For this reason, the adoption of Alternative Sports (AS), such as Cornerball, is proposed. Cornerball, a playful–sporting variant, combines elements of wall and rebound sports with a divided court. It is played in mixed-gender pairs within a 90-degree corner divided by a net at the center, with players alternating participation on each hit. This novel approach seeks to enhance student motivation, promote physical activity, and convey values in an engaging manner. The main objective of this paper is to design a didactic proposal for the implementation of Cornerball in PE classes. Its potential to foster active participation, coeducation, sports values, cooperation, and healthy lifestyle habits is highlighted. This new alternative sport emerges as an attractive and challenging option, contributing significantly to the evolution of Physical Education toward a more holistic and motivating approach.

## 1 Introduction

In recent decades, Physical Education (PE) has undergone modifications in its curricular components with the aim of evolving towards a more contemporary model capable of responding to the educational demands of the twenty-first century (1). Teachers must therefore continually adapt in order to meet the increasing demands of this new era, exploring innovative approaches with the purpose of enhancing students' motivation (2, 3). In this regard, one of the most powerful tools available to teachers for achieving pedagogical objectives is educational sport (4, 5).

Along these same lines, Jaquete & Ramírez (6) suggest that educational sport should focus on the transmission of values and equitable participation by all students, regardless of their motor competence, distancing itself from competitive or high-performance sport models (7). This position is supported by a substantial body of literature demonstrating that traditional sports often fail to motivate students due to their promotion of excessive competitiveness (8, 9).

Therefore, the Sport Education Model emerges as an ideal strategy in the context of teaching racket sports and sports initiation, as it allows students to be organized into stable teams, assume diverse roles, structure practice into seasons, and promote authentic participation and contextualized learning (10–14). In this line, the aim of this paper is to provide the educational community with a pedagogical intervention proposal based on a new alternative racket sport. This initiative seeks to introduce this sport into educational settings in a novel and transformative manner, using play as a fundamental educational resource to enhance students' learning and motivation.

## 2 Pedagogical framework

In response to these pedagogical demands, so-called Alternative Sports (AS) have emerged with the aim of promoting maximum participation (15), encouraging healthy lifestyle habits (16–20), and being more attractive, novel and motivating for students (5, 21). Furthermore, they seek to promote values and gender equality (22) and to facilitate inclusive participation through flexible rules that make sporting practice more accessible to all learners (23).

Accordingly, an innovative proposal is presented based on the Sport Education Model (SEM) (24, 25), to be implemented in Physical Education lessons. This model aims to replicate the structure of sport (pre-season, season and culminating event) within the school context and to bring students closer to the sporting domain, enhancing values and social responsibility (26), developing sport-specific skills and roles (27), and generating more meaningful learning environments in educational settings (28).

Moreover, racket sports are presented within Physical Education as a pedagogical approach of considerable educational value (29, 30), contributing to the improvement of students' motor skills and decision-making processes, as well as their affective and social skills (31, 52).

Cornerball is defined as a hybrid playful-sporting activity that combines wall sports and net or divided-court games. It is played in mixed pairs on a 90-degree corner court divided by a net, and participation alternates with each stroke, promoting equitable involvement among all players (32). This conceptualisation aligns with the Spanish educational framework, as Royal Decree 217/2022 (51) supports the curricular inclusion of alternative and non-traditional sport practices in Physical Education.

## 3 Learning environment

### 3.1 Setting

Emerging content and alternative sports have progressively gained a place within the educational curriculum, with the aim of transforming PE into a renewed, innovative and inclusive subject. In this regard, Cornerball is presented as a hybrid sport that combines elements of divided-court sports and wall-based sports (32).

### 3.2 Participants

The proposal presented in this paper is aimed at secondary education students aged between 12 and 16 years. A typical class consists of 24 students, organised into four groups of six. In the first session, the different roles are explained and then assigned at the beginning of each session. These roles are carried out when students are not participating as players.

This approach supports the inclusion of students with specific educational support needs. The flexible rules and cooperative structure of Cornerball allow adaptations to equipment, space, and game conditions. In addition, the rotation of roles within the Sport Education Model ensures that all students can participate according to their abilities, promoting equity, autonomy, and social inclusion.

### 3.3 Pedagogical format

With the aim of incorporating the teaching of Cornerball into Physical Education lessons, a range of methodological strategies are proposed. In this respect, a methodology based on modified or simplified games, mini-games, and simple game-based tasks is advocated in order to facilitate learning and understanding of the sport's tactical aspects, as suggested by Courel-Ibáñez et al. (33). Accordingly, particular emphasis is placed on key elements such as tactical awareness and students' motor autonomy.

The introduction of a novel sport enables all students, including those with lower skill levels, to develop technical-tactical competencies (34), as it promotes the resolution of motor problems by ensuring that all participants start from a similar level of prior knowledge of the sport. Furthermore, it is recommended to initially increase the height of the net and subsequently lower it progressively to the official height, with the aim of slowing down the pace of play. This approach facilitates students' reaction time and the recovery of strategic positions, in line with the recommendations of Thorpe (35).

Moreover, the sport initiation model selected in this proposal to teach Cornerball and to motivate students while consolidating their learning is the Sport Education Model (SEM), proposed by Siedentop (24, 36) and Siedentop et al. (25). This model seeks to simulate a sporting season within the educational context by organising students into stable teams (affiliation), thereby fostering a sense of belonging, promoting values of cooperation and group cohesion, and developing social skills and competencies. To this end, a range of rotating roles is assigned to each participant, including coach, team representative, referee, analyst, equipment manager, and fitness trainer (Supplementary Table S1).

In accordance with the Sport Education Model (SEM), Physical Education sessions for teaching Cornerball are structured as a “sporting season” (SupplementaryTable S2), divided into pre-season, season, and celebratory phases, with a schedule of training and competition designed to promote gradual learning progress.

During the pre-season (sessions 1–2), students become familiar with the rules, roles, and game dynamics through teacher-guided activities, using games and tasks aimed at familiarization with the equipment (ball, paddle, and paddle–ball combination) and following a progression consistent with the logic of the game.

During the season, training and competition alternate: sessions 3, 5, 7, and 9 are devoted to competition, with one team acting as host, while non-competing teams continue training. Sessions 4, 6, and 8 focus on training and are designed by the coach, using Supplementary Table S3 as a reference to develop technical–tactical skills and adapt tasks to the needs of the group.

The celebratory phase (session 10) includes the final competition and the awarding of prizes and badges, conducted in a league format to ensure the participation of all teams without eliminations.

Each session follows a consistent structure: a warm-up with games to familiarize students with the equipment (ball, paddle, and ball–paddle combination), a main part (competition or training), and a cool-down, ensuring coherence and safety. Coaches have access to a supplementary worksheet (Supplementary Table S3) with games and motor tasks that allows them to plan and adapt sessions according to the students' needs.

### 3.4 Equipment

The didactic resources that may be employed for the implementation of the sessions proposed in the teaching of this new alternative sport are as follows:

Installations: One sports hall or a similar space with corners free from obstacles. If four corners are not available (one for each group), all teams will take turns using the available corners. Meanwhile, the groups without access to a corner will carry out training tasks facing a single wall, under the guidance of the group’s “coach”.
Materials: 24 paddles, 12 small rubber balls, foam rubber balls of different sizes, cones, four corner nets, elastic bands, net supports, and related equipment. In contexts with limited material resources, Cornerball can be implemented using self-constructed equipment, enabling its adaptation to diverse school settings without compromising its pedagogical aims. The hybridisation of the Sport Education Model with student-designed materials further supports interdisciplinary work, fostering team identity, creativity, and responsibility through the creation of equipment, logos, and banners, as supported by recent research on student-made materials in Physical Education (37–39).

## 4 Results

The introduction of Cornerball in the educational context could potentially promote meaningful learning related to sports practice. As a hybrid sport combining elements of wall-based and divided-court sports, it would present a distinctive internal logic that might shape spatial relations, interaction with the playing object, and motor communication. Its game dynamics could foster decision-making, coordination, role assumption, and the management of uncertainty, thereby supporting the development of technical and tactical skills potentially transferable to other sociomotor sports.

When integrated within the Sport Education Model, it could allow for stable teams, diverse roles, and season-based practice, encouraging authentic participation and contextualized learning. This combination might enhance motor, social, and cognitive competencies, promote autonomy, and deepen game understanding. Supplementary Table S4 would illustrate how different parts of the session could be assessed using various assessment instruments and procedures. This evaluative approach would allow for the analysis of both learning outcomes and learning processes, reinforcing the educational, inclusive, and motivational nature of the proposal, and ensuring coherence with the pedagogical principles of Physical Education oriented toward the promotion of active and healthy lifestyles.

## 5 Discussion

This study aimed to provide the educational community with a pedagogical proposal based on a novel racket-based alternative sport, Cornerball, implemented through the Sport Education Model (SEM). SEM has been widely applied in school Physical Education and has demonstrated its effectiveness in promoting meaningful learning, motivation, and social development (18, 40, 53).

In response to contemporary educational demands, Alternative Sports (AS) have emerged as effective pedagogical strategies to foster student engagement, inclusivity, and equitable participation, addressing the limitations of highly competitive traditional sports (21, 22, 41, 42). Previous research indicates that SEM-based and AS interventions are associated with higher levels of enjoyment, motivation, prosocial behaviour, autonomy, and gender equity awareness among adolescents (38, 39, 43–46).

Similarly, racket sports have been shown to support motor, affective, and social development, while contributing to students' well-being through engaging and dynamic learning environments (30, 47–49, 54). Nevertheless, their implementation in school settings is often limited by contextual constraints such as insufficient facilities or teacher training (50).

Within this framework, the integration of Cornerball and SEM offers an innovative pedagogical approach that combines technical-tactical learning with social development and student motivation. Unlike other alternative sports commonly implemented in Physical Education, Cornerball is characterised by a distinctive internal logic that integrates wall-based and divided-court dynamics within a single playing space, generating unique spatial constraints that demand continuous perceptual, tactical, and decision-making adaptation.

A defining characteristic of Cornerball is the compulsory alternation of strokes between partners, which structurally limits individual dominance and promotes equitable participation, regardless of students' level of motor competence. Furthermore, the use of a 90-degree corner introduces unconventional perceptual and cooperative demands, fostering the development of spatial awareness, anticipatory skills, and coordinated action in ways that are rarely addressed in traditional or alternative net and invasion sports.

In addition, this sport promotes a distinct form of spatio-temporal development, arising from the possibility of the ball rebounding off two different walls, which significantly conditions the dynamics of play. The angle of rebound, the angle of striking, and the force applied determine the ball's trajectory, directly influencing variables such as speed, height, or deceleration, and thereby requiring continuous perceptual–motor adaptation from the participants.

## 6 Conclusion

This paper presents Cornerball, implemented through the Sport Education Model (SEM), as an innovative pedagogical alternative for school Physical Education. The proposal is theoretically grounded in contemporary educational approaches and is designed to foster student motivation, active participation, motor skill development, social interaction, autonomy, and critical reflection.

One limitation of the proposal is the specific spatial requirement, as the game requires a 90° corner for its implementation. Furthermore, although empirical evidence on its direct educational impact is not yet available, the framework offered in this study provides a basis for future research examining its effects across different educational contexts and age groups.

From a practical perspective, Cornerball constitutes a feasible and pedagogically coherent option for Physical Education teachers, as it can be integrated into existing curricula without the need for extensive material resources or prior student experience. Its adaptable rules and alignment with the Sport Education Model support inclusive teaching practices, enhance student engagement, and offer a practical tool for addressing motivational and social objectives in secondary education.

## Data Availability

The original contributions presented in the study are included in the article/Supplementary Material, further inquiries can be directed to the corresponding author.
