# Supplementary material for: Teaching cornerball: a didactic proposal based on the sport education model
Source: Front Sports Act Living. 2026 Mar 2;8:1784916. doi: 10.3389/fspor.2026.1784916 (PMC12989537; doi:10.3389/fspor.2026.1784916)
Supplement: Supplementary file 1 [file Table1.docx]

Supplementary Material

# Supplementary Table 1

| **Table 1:**  *Tasks assigned to each group role..* | |
| --- | --- |
| **CAPTAIN/COACH** | Acts as a role model for the rest of the team members. This is the only player authorised to communicate with the officiating team during competition. The captain/coach is responsible for planning training sessions aimed at improving the technical-tactical aspects of the sport, as well as for establishing team strategies during competitions. |
| **TEAM REPRESENTATION** | Is responsible for communication with other team representatives and with the teacher. Additionally, this role involves welcoming the teams during the competition day organised by each group. |
| **REFEREE** | Is responsible for knowing, applying and enforcing the rules of the game, as well as for organising competitions. Furthermore, the referee is in charge of recording the score during matches, together with the referee from the opposing team. |
| **ANALYST** | Records performance-related data concerning their own team and gathers information about opposing teams. This role is also responsible for conducting co-assessment processes of both individual players and the team. |
| **EQUIPMENT MANAGER** | Is responsible for distributing, collecting and ensuring the appropriate management of the equipment required for each session. |
| **FITNESS TRAINER** | Is responsible for leading the warm-up activities for their team prior to practice or competition. |
| **Note:** Author’s own elaboration. | |
