# Supplementary material for: Teaching cornerball: a didactic proposal based on the sport education model
Source: Front Sports Act Living. 2026 Mar 2;8:1784916. doi: 10.3389/fspor.2026.1784916 (PMC12989537; doi:10.3389/fspor.2026.1784916)
Supplement: Supplementary file 2 [file Table2.docx]

Supplementary Material

# Supplementary Table 2

| **Table 2.**  *Sequencing of the proposal for teaching Cornerball.* | | | | | | |  |
| --- | --- | --- | --- | --- | --- | --- | --- |
|  | **PRE-SEASON** | | **SEASON** | | **CELEBRATORY** | |  |
| **Lesson 1:** | | Pre-season training 1 | |  | |  | |
| **Lesson 2** | | Pre-season training 2 | |  | |  | |
| **Lesson 3** | |  | | Matchday 1 | |  | |
| **Lesson 4** | |  | | Training session 1 | |  | |
| **Lesson 5** | |  | | Matchday 2 | |  | |
| **Lesson 6** | |  | | Training sesión 2 | |  | |
| **Lesson 7** | |  | | Matchday 3 | |  | |
| **Lesson 8** | |  | | Training session 3 | |  | |
| **Lesson 9** | |  | | Matchday 4 | |  | |
| **Lesson 10** | |  | |  | | Final event | |
| *Note:* Author’s own elaboration. | | | | | | |  |
