# Supplementary material for: Teaching cornerball: a didactic proposal based on the sport education model
Source: Front Sports Act Living. 2026 Mar 2;8:1784916. doi: 10.3389/fspor.2026.1784916 (PMC12989537; doi:10.3389/fspor.2026.1784916)
Supplement: Supplementary file 3 [file Table3.docx]

| **Tabla 3.**  *Supplementary materials for teaching Cornerball.* | |
| --- | --- |
| **WARM-UP** | |
| 1. **Keep the ball in the air**: Players keep the ball in the air using the paddle for as long as possible. 2. **Partner passes**: Players gently hit the ball back and forth with a partner, focusing on control. 3. **Free ball control on the paddle**: Players move freely while balancing or controlling the ball on the paddle. 4. **Target hitting**: Players hit the ball with the paddle aiming at different targets. 5. **Bounce and hit**: Players let the ball bounce once and then hit it with control using the paddle. | |
| **MAIN PART** | |
| **Task 1 – Fundamental strokes in trios:**  Participants form triangles, each with a racket and a ball. They practise bouncing and hitting the ball to each teammate, aiming to maintain continuous strokes with a bounce. | 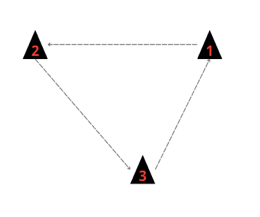 |
| **Task 2 – Wall strokes:**  Trios stand in front of a wall with one ball and three rackets. Players hit the ball alternately against the wall, starting with drives and then incorporating forehand and backhand strokes. | 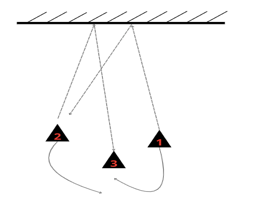 |
| **Task 3 – Wall challenges:**  Specific areas on the wall (circles, lines, numbered points) are designated as targets. The group must collectively hit all the targets. | |
| **Task 4 – Continuous play between two walls:** Teams maintain a continuous play by hitting the ball alternately against both walls from the back of the court, without a net. | 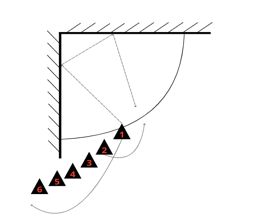 |
| **“The Strap” game:**  The team occupies the service zone. After serving, the server runs to the opposite side while a teammate takes their place. The receiving player does the same after hitting, maintaining a continuous rally. | 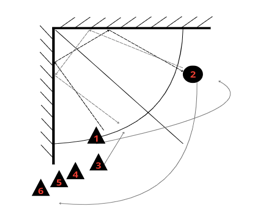 |
| **“The Wheel” game:**  Trios on each side of the net maintain a continuous rally, switching sides of the court after each hit. | 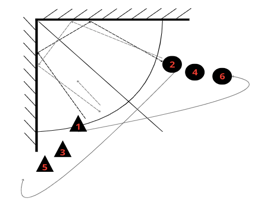 |
| **“The Rubber” game:**  A starting zone is marked. Each player, after hitting the ball against both walls, runs to the corner and returns to the start. The remaining teammates keep the ball in play with a single bounce. | 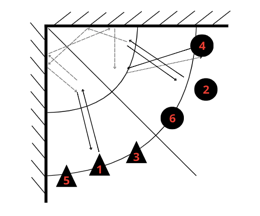 |
| **“The co-evaluation”:**  Players are divided into pairs. The aim is to maintain continuous play, but each time they hit the ball to different zones on the wall, they score more or fewer points. The remaining students who are not playing act as analysts (performing initial co-evaluation), referees, or coaches, providing feedback and correcting possible errors. | |
| **Note:** Author’s own elaboration. | |
