# Supplementary material for: Teaching cornerball: a didactic proposal based on the sport education model
Source: Front Sports Act Living. 2026 Mar 2;8:1784916. doi: 10.3389/fspor.2026.1784916 (PMC12989537; doi:10.3389/fspor.2026.1784916)
Supplement: Supplementary file 4 [file Table4.docx]

Supplementary Material

# Supplementary Table 4

| **Table 4.**  *Assessment instruments and procedures* | | | |
| --- | --- | --- | --- |
| **Instrument** | **Assessed dimensions** | **Evaluator** | **Timing** |
| Observation checklist | Participation, rule compliance, role involvement | Teacher | Throughout sessions |
| Rubric | Technical-tactical execution, decision-making, cooperation | Teacher / Peers | Training and competition |
| Peer-assessment sheet | Teamwork, fair play, communication | Peers (analyst role) | Competition sessions |
| Self-assessment questionnaire | Motivation, perceived competence, responsibility | Students | End of season |
| Team reflection log | Team organisation, strategy, role performance | Team (coach/captain) | Post-session |
